# Case Report: “One Unhealth” approach on fatal consequences of a fire in an animal hoarded home

**DOI:** 10.3389/fvets.2026.1719824

**Published:** 2026-02-05

**Authors:** Louise Bach Kmetiuk, Jéssica Santos da Silva, Ricardo Guedes Correa, Claudia Cristina Brositti Terzian, Vivien Midori Morikawa, Alexander Welker Biondo

**Affiliations:** 1 Department of Veterinary Medicine, Federal University of Paraná State, Curitiba, State of Paraná, Brazil; 2 Department of Collective Health, Federal University of Paraná State, Curitiba, State of Paraná, Brazil; 3 City Secretary of Environment, Curitiba, State of Paraná, Brazil

**Keywords:** animal welfare, case report, hoarding disorder, One Health, public health

## Abstract

Animal hoarding is directly associated with impaired human and animal health. In this study, we describe the tragic end of a household fire involving an individual who was hoarding animals. In 2013, the city fire department and the Animal Protection Department of Curitiba were called to respond to a house fire at the residence of an elderly woman with around 70–80 dogs. She had a long-standing diagnosis of animal hoarding behavior and was involved in multiple ongoing judicial processes for animal neglect and cruelty. According to witnesses, the most likely cause of the fire was the lighting of candles indoors, as electricity and water had been cut off on the premises for at least 3 years. A woman in her late 60s fled the scene, but 43 dogs were found dead due to asphyxiation and fire in the main bedroom, with another seven dead dogs found beneath the house. A total of 29 dogs were recaptured after they escaped into the streets during the fire. Despite their infrequency, incidents of household fires involving animal hoarding require increased attention from public health authorities to prevent them effectively. The consequences and implications of these incidents are discussed, and a protocol for preventive strategies is proposed.

## 1 Introduction

Animal hoarding behavior is characterized by the accumulation of a high number of animals and resistance to their disposal among other possessions, often alongside a failure to provide adequate care for the animals (1). This represents one of the worst-case scenarios for the World Health Organization (WHO)'s “One Health” approach, with human, animal, and environmental health often at high risk. Although animal hoarding is considered a special manifestation of hoarding disorder, some authors argue that it should be classified as an independent nosological entity. Animal hoarding behavior may also be a clinical display of other underlying mental disorders, including addiction, dementia, and focal delusions (2). The prevalence of animal hoarding is 3.71 cases per 100,000 inhabitants in Brazil, compared with 0.8 in the United States and 1.78 in England (2).

More generally, hoarding behavior is associated with a high risk of fatal fire accidents (3). For example, in Australia, although hoarding accounts for only 0.25% of all residential fires, it is responsible for 24% of preventable fire fatalities (3). This was because hoarding fires mostly involve elderly individuals, requiring a higher provision of resources than average residential fires.

More specifically, regarding animal hoarding, although this often worsens the risks to both human and animal life within the scope of One Health, studies focusing on the households themselves as a risk factor are currently lacking. This includes investigating the potential origins of such tragedies, including objects (e.g., lit candles) falling on individuals or animals, which can ultimately lead to a house fire.

The present study reports a household fire involving individuals who hoard animals and describes the events before, during, and after the accident. The case involves an elderly woman who hoarded animals and regularly used candles inside her home because her electricity and water services had been cut off 3 years earlier. This study details how this led to a house fire that killed 50 of her dogs, highlighting the risk factors of major accidents involving vulnerable individuals and populations.

## 2 Case description

**Figure 1:**
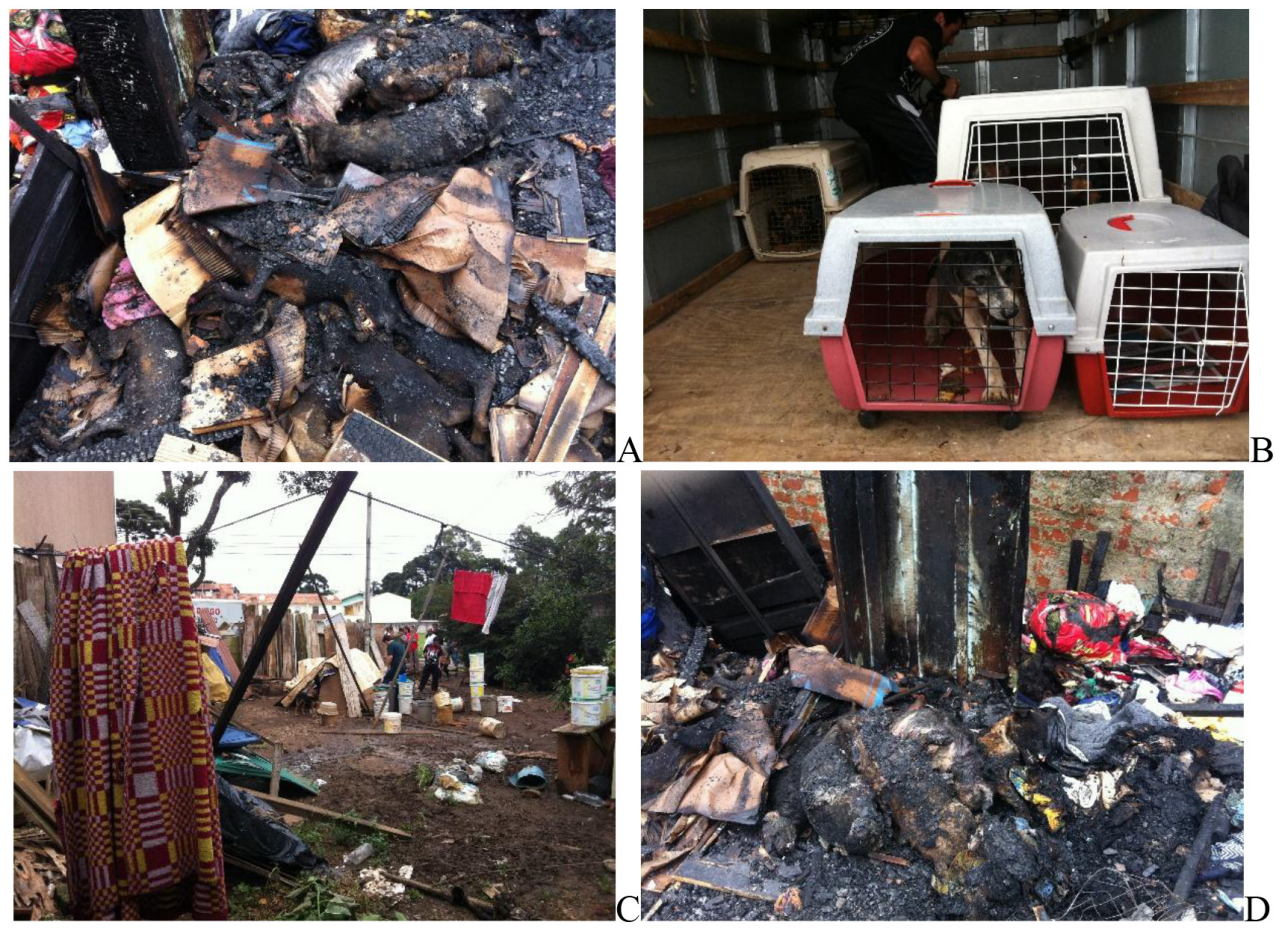
Photos of burned dog remains, taken in the house fire aftermath, stacked as observed in human fire accidents, leading to painful death by asphyxiation **(A)**. All dogs outside the house survived without burnings and were rescued **(B)**. Unhealthy conditions daily suffered by hoarding dogs **(C)**. Burned dog remains found after the house fire **(D)**.

This case report pertains to an incident that occurred in 2013, when we served as the Director and Vice Director of the Curitiba City Animal Welfare and Control Service. In April of that year, we were contacted by firefighters and animal protection groups to assist with a case of a house fire involving an animal hoarder (Figure 1). Additional information was obtained from city and state records due to ongoing legal prosecution involving the individual responsible at that time. Psychiatric or other medical records were not available. This study follows a 10-year statutory period during which we waited to report on our findings, and we have omitted all personal information that could reveal the identities of those involved.

The incident occurred in Curitiba, the capital of Paraná State, which is ranked the 8th most populous city in Brazil with approximately 1.8 million inhabitants. It has the 13th highest gross domestic product and the 10th highest municipal human development index (HDI) out of 5,565 municipalities in Brazil (4). Based on the city's complaints statistics, Curitiba has 6.45 confirmed hoarding cases per 100,000 inhabitants, of which 42.5% involve objects, 36.3% animals, and 21.2% involve both (5). Interestingly, the metropolitan area of Curitiba recently reported a very high level of hoarding case identification, possibly due to better detection and monitoring by public services (6).

In the early hours of Sunday morning on April 17, 2013, the Animal Protection Department of Curitiba City was called to assist the city fire department in responding to a fire in a wooden house belonging to a woman in her late 60s with 70–80 dogs, long-time diagnosed with animal hoarding and who was facing an ongoing judicial process for animal neglect and cruelty. Upon arrival, the house windows and doors were found closed, with 43 dead dogs in the main bedroom due to asphyxiation and burns from the fire, and another seven dead dogs underneath the house. The remaining 29 dogs in the yard fled into the neighboring streets. According to witnesses, the woman had been using candles for light because electricity and water had been cut off 3 years earlier. The woman escaped the house through the window, without helping the dogs. The escaped dogs (20 adults and a female with eight puppies) were recaptured and sent to a private animal shelter for adoption.

Prior to the fire, publicly filed complaints over several years had prompted a series of visits, inspections, justice orders, and interventions by public services. Starting on December 13, 2010, the city service for animal protection visited the property in response to complaints of animal cruelty. The owner was not at home at the time of inspection, but 10 adult mixed-breed dogs were observed over the fence in the front yard in unhygienic conditions, mud, and rubble, as well as food waste and feces. A warning was issued for the household. New inspections were performed thereafter due to new complaints on June 13 and July 20, 2011, during which the woman was present. Forty dogs were found in the front yard, some showing signs of weight loss and generalized infection. The owner reported that the electricity and water services were cut off due to nonpayment, and that she used donated gallons of water and candles for light at night inside the house. Although the dogs were in poor condition and showed signs of neglect, the city service had no shelter spaces at that time, and the dogs were kept and treated on-site. On January 9, 2012, another complaint of animal cruelty was made, with 80 dogs found upon inspection, despite the owner claiming only 48. Some presented bad odors, scabies, and other diseases, and all were treated at the property. On July 25, 2012, a complaint was made concerning dogs with mange and intestinal worms, as well as the owner's aggression toward the dogs. The dogs were treated, city sanitary services were provided, and the State Environment Public Prosecutor's Office ordered the adoption of all animals. On August 20, 2012, another complaint was lodged concerning 80 dogs with mange and other diseases, barking, and emitting an unpleasant odor. The dogs were treated for mange, worms, fleas, and ticks over the following days, and adoption was recommended. On November 8, 2012, another complaint was made regarding a lack of sanitation and poor personal and animal hygiene. The dogs were vaccinated, dewormed, and administered an antiflea treatment. As the owner refused to give the dogs up for adoption, the city's social assistance service was contacted. More complaints were filed on January 9 and 21, 2013, with no changes in the animals' or the owners' situations. During this period, the house lacked electricity and water services.

Despite numerous efforts over the years, the woman refused psychiatric evaluation provided by the city Secretary of Health, even when mandated by the State Justice Court, using her daily duties and fragile health conditions as justifications for missing appointments. However, city psychiatrists also refused in-loco visits due to the risk of exposure to unhealthy conditions (against occupational health standards), impairment of proper professional assessment in such an environment, and the impracticality of door-to-door visits to hundreds of suspected hoarding cases. A definitive diagnosis and monitoring would require multiple medical consultations over a relatively long period of time.

In May 2012, the State Court issued an order prohibiting the woman from acquiring more dogs. In August 2012, a public civil action was initiated and was at the stage of the final decision and sentence when the house fire occurred. On February 19, 2013, the State Justice Court requested a preliminary order and awaited the decisions when the fire occurred. The woman was considered criminally unimputable due to her mental disorder and was not charged for neglect, the fire, or animal cruelty, but instead sent for further psychiatric treatment. All surviving dogs were taken to local volunteers and animal shelters.

According to the Brazilian Criminal Code, Law 7,209 of July 11, 1984, in its Article 26th: “persons with mental disorders or incomplete mental development or retardation at the time of action or omission, fully incapable of understanding the illicit character of the facts, are exempted from charges.” Many other countries have also considered that mentally ill individuals who commit nonviolent crimes should be treated instead of punished, with hospitalization and eventual discharge (7).

However, another cruelty complaint was made in early 2018 about the same woman at a different address. During the inspection visit, she voluntarily told city personnel about her previous house fire and the deaths of 50 dogs, which she stated was caused by an unattended candle. She admitted owning 19 dogs in this new address but denied entry to inspectors. Electric power was cut off again because of nonpayment. At this time, she allowed four male unattended individuals to enter the home.

She moved to another house in early 2019, where another complaint led to a city inspection and the removal of six dead dogs in different stages of putrefaction. In September 2020, in her early 70s, she experienced another house fire, resulting in the death of 10 dogs and the rescue of 20. Following the police investigation, two men were arrested and criminally charged for arson, bodily injury, and animal cruelty; one was identified as the house owner, who likely started the fire due to overdue rent payments.

After the second fire, she moved to her sister's house, who informed by phone in September 2023 that the woman had only one dog, spayed by the city services, and was living alone at an undisclosed address.

## 3 Discussion

In this case, the house was made of wood and provided ample fuel for a fire, along with clothes, furniture, and other flammable items. As fire requires fuel, a spark, heat, and oxygen, hoarding represents a significant fire risk (3). The disorganized stacking and accumulation of possessions provides fuel that is often highly flammable and can spread quickly, including piled clothes, old papers (newspapers, books, and magazines), and plastic (empty bags and used packages) (3).

### 3.1 How this case fits within the literature

Despite few reports on house fires associated with hoarding behavior, fire hazards account for approximately 67% of hoarding complaints in Massachusetts, United States, with 6% directly related to resident deaths (8). In Vancouver, Canada, 80% of hoarding complaints involved fire code violations, forcing Fire and Rescue Services to build a coordinated hoarding response based on fire inspections, achieving satisfactory results in 94% of cases without prosecution (9). A study in England found that 34% of households with hoarding behavior presented fire and environmental risks, with a high economic burden on housing providers and emergency services compared to the general population (10). Nonetheless, several cases of general hoarding may become public only when a crisis or tragedy strikes, requiring the involvement of multiple health-service professionals (11).

The present case was not the first in Brazil (12). A previous fire was report in São Paulo City, Brazil, and involved an elderly woman who was found unconscious in the living room near lighted candles and was attended by firefighters but died at the scene due to severe burn wounds and smoke inhalation. Similar to the present study, 10 of the 25 dogs died of asphyxiation and burns. Cases have also been described in the United States. In 2022, the son of an 86-year-old woman died along with 31 of their 37 dogs in Phoenix, Arizona, with the remaining dogs rescued by the Arizona Humane Society (13). In 2023, 43 Chihuahua dogs belonging to an owner with compulsive hoarding were rescued in Indiana after a fire scorched the property, leaving the dogs to run wild in unsanitary conditions; no one was burned or hurt in the incident (14). In 2023, a fire in an elderly man's house caused by a reignited smoldering stove killed 25 of his 45 dogs, pushing county commissioners and the local Humane Society to search for guidance on dealing with animal hoarding (15).

Isolated elderly individuals with hoarding behavior who own of many dogs may be more predisposed to physical accidents, including stumbling, falling, and dog bites (16). As observed herein, the cognitive difficulties that give rise to animal hoarding can result in unintentional accidents (17), including house fires due to forgotten lit candles and overheated stove pans. In fact, 31–93% of individuals who hoard animals do so under unsanitary conditions (2). A review of the literature shows that among surveyed residences, the type of possessions accumulated by animal hoarders is garbage (60%), litter (70%), and dispersed animal feces and urine (40%). House fires were only mentioned once in our review of the literature, with approximately 17% of households considered unsafe for living due to clutter and the risk of fire (2).

Hoarded possessions can impair passageways during a fast-spreading house fire, for the homeowner as much as for firefighters and other first responders, posing an additional risk for emergency personnel (3). According to witnesses, the woman in the present report fled through a narrow window space without opening the front door or large windows, leaving her dogs locked inside the house, which led to this tragic accident. Not surprisingly, older adults with hoarding disorders may have increased medical comorbidities and may be more prone to fires, in addition to poor nutrition and self-hygiene, compared to non-psychiatric elderly individuals (18).

A recent systematic review evaluating 538 animal-hoarding individuals found that most are middle-aged (62.5 years old), unmarried females with poor self-hygiene, living alone (63.8%) in urban areas under unsanitary conditions (65.2%) (2). As older individuals are three-fold more predisposed to hoarding behavior than younger adults, vulnerability may be worsened by elderly comorbidities and daily difficulties (19). Hoarded animals typically include dogs and cats, often acquired through uncontrolled breeding; they often have disease, injuries, or behavioral problems, and animal carcasses are found in 60% of cases (2). A previous review describing cases of ownership of multiple dogs in New York City represented the first scientific publication on animal hoarding. Despite this, at present, scientific studies focusing on the impact of house fires on animal hoarding are lacking. As a result, these tragic events are typically reported only through news coverage. Fires in the homes of animal hoarders have been reported in Brazil, Mexico, the United States, England, Australia, and Spain (20–28). In most cases, an isolated elderly person was found injured or dead in the household, and the rescued animals were temporarily sheltered, with calls and contacts for prompt adoption, alongside long-term neighborhood complaints.

In addition to psychiatric conditions, individuals with a hoarding disorder (HD) were more likely to report a lifetime history of cardiovascular/metabolic conditions, including diabetes and hypercholesterolemia. Those with HD and subclinical HD were also more likely to report chronic pain and sleep apnea than non-HD participants. Thus, the medical burden of HD is higher than that of the general population, and increases with increased severity, particularly in the case of cardiovascular/metabolic dysfunction, autoimmune, sleep, and pain-related medical conditions (29).

Individuals with HD present several risk factors, with cognitive impairment among them. However, the literature lacks consensus regarding cognitive functioning in HD. A previous study proposed a model of HD that included several risk factors that are thought to increase the likelihood that HD will develop, including genetic predisposition, abnormal brain structures, environmental factors (e.g., traumatic life experiences), neuroticism, and cognitive function impairments (30). While a previous review focused on evidence of difficulties sustaining attention, motor inhibition, and organization, the literature on deficits in other areas of executive function is wide ranging, limiting the extent to which firm conclusions can be drawn (31). A previous study conducted in 178 adults found no reliable differences in cognitive functioning between hoarding and healthy individuals. Furthermore, specific hoarding symptoms were not found to be correlated with cognitive functioning. Another study examined the subjective appraisal of the cognitive abilities and emotionally-laden cognition of individuals with HD, reporting dissociation between perceived and objective functioning in HD (32). No association was observed between self-reported memory difficulties and objective performance on verbal or visual memory tasks. Self-reported problems with attention were associated with objective attentional performance, although this relationship was partially accounted for by anxiety symptom severity (33). Taken together, the literature suggests that individuals with HD often exhibit poor or absent self-reflection, which can contribute to the recidivism of behaviors.

Although the case reported herein was a classic case of animal hoarding, no psychiatric report was available. Such failures in psychiatric assessment and official reports provide a basis for developing protocols specifically targeting animal hoarding, including monitoring, mitigation, and resolution.

### 3.2 Possible explanations for animal hoarding

Recent surveys have defined animal hoarding as a mental disorder that may be triggered by single or multiple traumatic events, mostly affecting middle-aged women in their fifties who live alone and experience a high level of social and health degradation, as described in a study on 29 Italian cases of animal hoarding (34). The study revealed serious signs of animal neglect, including dehydration, malnutrition, physical injuries, and behavioral disorders. Because of the sudden rescue and removal of dogs from the firehouse area by volunteers to multiple destinations, no clinical or behavioral examination of the affected dogs was conducted (34).

Owing to the high risk of fire in the United States and Canada, the households of individuals with HD represent a public health threat, both to the owners themselves and to the neighborhoods through infestations and unpleasant odors (35). In these scenarios, a hoarding response task force that combines fire prevention and public health services may be an effective tool for mitigating, controlling, and preventing animal hoarding. A recent study in England suggested focusing on risk stratification and employed a widespread approach to hoarding cases in general to address the complexity of health and social care issues within the community (10).

### 3.3 Consequences of animal hoarding

By concomitantly impacting human, animal, and environmental health, HD is considered a classic example of interconnectedness requiring the WHO's “One Health” approach. Community-based approaches and increased awareness may be required to detect, prevent, and respond to animal hoarding (36). Longstanding interventions are required to tackle these cases due to the time-consuming legal efforts often associated with HD, which are nevertheless often ineffective in both veterinary and sanitary aspects due to the unwillingness of affected individuals to cooperate and the high likelihood of relapse (37).

Cases like that of an isolated elderly woman almost dying in a house fire, along with several dogs, highlight the human impact of HD. This disorder mainly affects elderly, reclusive, and isolated individuals with comorbidities who are infrequent users of medical services. The latter makes these individuals continuously at risk of illness, trauma, and death. Animal hoarders are further endangered by owning numerous starved dogs that may feed on their owners' dead bodies (38).

Animal hoarders put the health of their animals at risk, as highlighted by our case: dozens of dogs owned by an elderly individual with HD burned to death. These animals suffer under confined conditions, often lacking veterinarian attention and a nutritional diet. Dogs under these conditions often lack vaccinations for preventable species-specific (parvovirus and distemper) and zoonotic (leptospirosis and rabies) diseases, a fact that could not be ruled out in this reported case. To this end, protocols and guidelines need to be established for the effective mitigation, control, and prevention of animal hoarding.

In addition to human and animal health, environmental health is also at risk in these cases, with house fires being one result of the unsanitary and unsafe environmental conditions caused by HD. Piles of waste pose an environmental hazard and restrict movement inside houses, leading to tripping and falling, but also to the spread of fire. Unpaid bills cause electricity and water to be disconnected, leaving isolated residents vulnerable to foodborne, vector-borne, and zoonotic diseases, ultimately leading to both human and animal suffering and fatal outcomes.

A limitation of the present study is that very few details are available on the clinical and behavioral aspects of hoarded dogs. As a result, clinical and laboratory assessments were not performed as established by the case report guidelines (CARE) (39). Also, the prosecution process referred to an animal hoarding diagnosis that was made. However, the medical report and correspondent physician were undisclosed (Supplementary file 1). Animal hoarding is considered a multifactorial and complex issue, with equally complex solutions that are mostly mitigative. Effective problem-solving and definitive resolutions are likely to require a multidisciplinary task force involving human and animal healthcare professionals to address the unhygienic and unsanitary conditions of individuals with HD and their animals. Including representatives of public safety, city inspectors, and firefighters is also necessary to restore household safety in these cases.

### 3.4 Protocols to intervene in cases of animal hoarding

This case report serves as a warning to public services and authorities on the importance of monitoring populations for HD, with elderly individuals living alone with several dogs in unsanitary conditions being the main demographic affected. These individuals not only pose risks to themselves, but also to their household residents (if any), neighbors, relatives, and friends. Moreover, this study highlights the need for an interdisciplinary approach involving public services and other stakeholders to prevent future tragic outcomes. Finally, this report provides a foundation for the development of preventive protocols to address cases of animal hoarding. The impact of these cases on human health (elderly mental illness and loneliness), animal welfare (starvation, cruelty, and abandonment), and environmental safety (household degradation and impairment) amounts to a “One Unhealth” approach, ultimately leading to the death of both individuals with HD and their animals (38).

The Singular Therapeutic Project (STP) is considered an important tool by the Brazilian Ministry of Health to assist mental health professionals in patient planning, assessment, and implementation, respecting the vulnerability and uniqueness of each case resulting from complex psychological distress, as observed in the hoarding behavior of the individual reported herein (40). Briefly, STP generally comprises four stages, starting with patient diagnosis and situational analysis of the physical, psychological, and social aspects, which may include mapping potentialities, resilience, vulnerability and risks, beliefs, interests and desires, relationships, and social support from family, work, and neighborhood. The next stage focuses on short-, medium-, and long-term objectives and actions, involving discussions and negotiations with the patient in a collective decision-making process. Then, responsibilities are assigned, and patient relationships are established, including the STP reference case professional, primary care team, and family. Finally, as the case evolves and progresses, it may require reassessment or new objectives (41). Despite demanding significant time and effort from a professional task force, the STP protocol is widely considered a successful and effective approach, ensuring high-quality care and accountability across a wide range of cases. In cases of animal hoarding, specifically, the STP approach may be a good option for balancing human, animal, and environmental health, in line with the “One Health” approach (42).

Ultimately, this study demonstrates the need for increased funding dedicated to cases of animal hoarding, particularly to improve the living conditions of animals and prevent such dire outcomes in the future (3). According to Sigmund Freud, a house may classically represent one's sense of self and subconscious mind (43); thus, “fire in the house” may indicate an internal turbulence mirroring real-life worries. The case presented herein may be considered a real-life consequence of the inner turmoil associated with HD, a mental disorder that can lead to animal hoarding.

This report of a house fire involving an elderly individual who hoarded animals highlights the urgent need to develop a specific protocol for HD based on the STP guidelines and the One Health approach. This will involve public services, including medical and veterinary services, to identify, inspect, and monitor cases of animal hoarding as a basis for accident and fire prevention. This study serves as a warning to public services and authorities, as well as to the relatives, friends, and neighbors of individuals with HD, on the importance of monitoring them, particularly elderly individuals living alone with several dogs in unsanitary conditions.

## Data Availability

The original contributions presented in the study are included in the article/Supplementary material, further inquiries can be directed to the corresponding author.
