# Supplementary material for: Case Report: “One Unhealth” approach on fatal consequences of a fire in an animal hoarded home
Source: Front Vet Sci. 2026 Feb 5;13:1719824. doi: 10.3389/fvets.2026.1719824 (PMC12916378; doi:10.3389/fvets.2026.1719824)
Supplement: Supplementary file 1 [file Supplementary_file_1.docx]

Supplementary Material

# Supplementary Material. Prosecution process of animal hoarding diagnosis


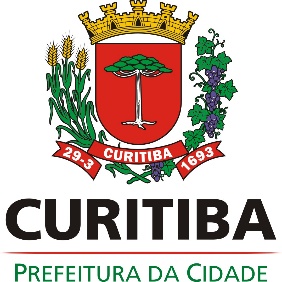


City Secretary of the Environment

Curitiba City Animal Welfare and Control Service

Rua Pres. Faria, s/nº

80020-290 Curitiba PR

Tel 41 3350-9920 / 3350-9940

Fax 41 3350-9941

e.mail: zoo@smma.curitiba.pr.gov.br

www:curitiba.pr.gov.br

Proposal No.: 6200062 / 2013

Type: Request for Information

Summary: Requests information about the case involving animal hoarder Patient name.

Text: Requests the Board, in accordance with the regulations, to forward a letter to the Mayor, requesting the following information:

1. List of all complaints filed with the Municipal Government against Ms. Patient name.;

In the early morning of March 17, 2013, Sunday, there was a serious fire at the home of Ms. Patient name., located at Patient address.

There were several reports of animal abuse against Patient name, and several inspections were carried out at the location, as described below:

As per the request contained in Official Letter No. 2445/2010 - PP MPPR-0046.10.000299-0, Public Prosecutor's Office of the State of Paraná, an inspection was carried out on 12/13/2010. The person responsible for the property was not at home, and from the gate it was possible to verify the presence of approximately ten mixed-breed dogs, adults, males and females, in precarious hygiene conditions, with the presence of mud, a large amount of debris, food scraps and animal feces. The dogs were loose, chained up, or sheltered in improvised kennels made of wooden boards and recycled plastic bags. Some dogs had no shelter to protect themselves from the cold, rain, or sun. The barking of other dogs could be heard, which were probably inside the house.

In response to the aforementioned official letter, new inspections were carried out on June 13, 2011 and July 20, 2011. In addition to the inadequate environmental conditions previously verified, the presence of approximately 40 (forty) mixed-breed dogs, adults and puppies, males and females, was verified. Some dogs were thin and others presented with generalized skin infection. According to the owner of the dogs, there was no water supply from Sanepar, nor electricity. She received donations of water jugs from neighbors. The situation of the animals was discussed and analyzed in conjunction with the Municipal Health Department, through the Center for Zoonosis and Vector Control, and although it was verified that the animals were in a situation of mistreatment, the municipality did not have physical facilities to house them.

On January 9, 2012, a complaint of animal abuse was filed (protocol 004.092.697) regarding animals with mange and foul odor, with 80 dogs presenting mange and other diseases. An inspection was carried out, where the owner only mentioned 48 dogs and did not allow entry into the house. Animals with mange were observed, and treatment was scheduled for them.

In the complaint dated July 25, 2012 (protocol 004.388.356), a request was made for an inspection of animal abuse, with injured dogs, skin wounds, and mange, and the resident abusing the dogs. The animals were medicated against mange and worms. The situation was monitored by the health surveillance agency. The person responsible was summoned by the Public Prosecutor's Office to arrange for the adoption of the animals.

On August 20, 2012, a complaint was filed regarding the case of Ms. Patient name (protocol 004.425.992) alleging at least 80 animals with mange and other problems, causing discomfort in the neighborhood. The animals were medicated against mange, worms, fleas, and ticks on August 22, 2012, and the adoption of the animals was recommended. In response to the animal abuse complaint filed on November 8, 2012 (protocol 004.541.045), an inspection was conducted and the premises were found to lack hygiene and sanitation. Some of the animals had previously been assisted by the Animal Defense and Protection Network, vaccinated, treated, and returned to Patient name, who refused to put the animals up for adoption. Given the circumstances, the protocol was forwarded to the Social Assistance Foundation (FAS) for assistance in approaching the resident.

In the complaints of mistreatment of 09/01/2013 (protocols 004.631.943 and 004.632.981) and 21/01/2013 (protocol 004652098) reporting the same inadequate situations in which the animals lived, an inspection was carried out and a new notification was issued for the owner to adapt the place, promote the adoption of the animals and not collect new animals.

Parallel to these complaints, in May 2012 an injunction was issued ordering Ms. Patient name not to collect new animals. Since August 2012, a Public Civil Action has been pending in the 23rd Civil Court, which is currently in the sentencing phase (case 22917-72.2012.8.160001). There was also a request for an injunction on February 19, 2013, for the removal of the animals against the owner's will. This injunction is awaiting a judicial decision.

As per the aforementioned report, it can be inferred that Mrs. Patient name actions are characteristic signs of animal hoarding syndrome, as described in the book Veterinary Forensics: Animal Cruelty Investigations, Merlinda Merck, 2007.

According to witnesses, in the early morning of the fire, the owner of the animals was using candles for lighting, as her residence was without electricity, which was the likely cause of the accident. Ms. Patient name did not provide assistance to the animals, which died of asphyxiation and charring inside the residence, totaling 43 deaths. 29 dogs were rescued alive, including 1 female with 8 puppies and 20 adult animals that were trapped outside the residence. These animals are under the care of the Amigo Animal Association, the Curitiba Animal Protection Society, and the Amigo de Raça animal protection group, and will be available for adoption at an opportune time.

This was the report made by the Department of Fauna Research and Conservation about the incident.

2. During the inspections, was it found that there was a lack of minimum conditions for maintaining the animals under the offender's care?

As verified in the report above, the animals' biggest problems were mange and lack of general care, which did not constitute minimum conditions in legal terms. Furthermore, according to the inspection reports, the team was not allowed entry on several occasions. Even so, free assistance was offered to the animals, and official letters were sent to the Public Prosecutor's Office on at least two occasions.

3. If the answer to the previous question is affirmative, what are the reasons why the provisions of Article 16, Paragraph 3 of Municipal Law 13.908/2011, in verbis, have not been complied with?

Below is the full text of the two official letters sent to the Public Prosecutor's Office:

TECHNICAL VISIT REPORT 01 (Curitiba, December 13, 2010)

Reference: Official Letter No. 2445/2010 - PP MPPR-0046.10.000299-0. Public Prosecutor's Office of the State of Paraná.

As requested in the aforementioned official letter, we inform you that on December 13, 2010, an inspection was carried out at a residence located at Patient address.

At the time of the visit, the person responsible for the property was not at home. From the gate, the following situations could be observed:

- Presence of approximately ten mixed-breed dogs, adults, males and females;

- The hygiene conditions of the place are precarious, with the presence of mud, a large amount of debris, food scraps, and animal feces. The ground is dirt, which makes adequate cleaning impossible;

- The dogs were loose, chained, or sheltered in makeshift kennels made of wooden planks and recycled plastic bags. Some dogs have no shelter to protect themselves from the cold, rain, or sun;

- Barking from other dogs could be heard, which were probably inside the house.

TECHNICAL VISIT REPORT 02 (Curitiba, July 25, 2011)

Reference: Official Letter 2445/2010 - PP MPPR-0046.10.000299-0

As requested in the aforementioned official letter, we inform you that inspections were carried out on June 13, 2011 and July 20, 2011 at the residence located at Patient address.

During the inspections, the following situation was observed (photos attached):

- Land in precarious hygienic conditions, with the presence of mud, a large amount of debris, food scraps and animal feces;

- Presence of approximately 40 (forty) mixed-breed dogs, adults and puppies, males and females; The dogs were found loose, chained, or sheltered in makeshift kennels made of wooden planks and recycled plastic bags, with some lacking shelter to protect themselves from the cold, rain, or sun;

- Some dogs were thin, and others had generalized skin infections;

- There were few water bowls available for the animals.

According to the owner of the dogs, there is no water supply from Sanepar (the water company) or electricity in the area. She receives donations of water jugs from neighbors.

The animals' situation was discussed and analyzed in conjunction with the Municipal Health Department, through the Zoonosis and Vector Control Center, and although it was found that the animals were being mistreated, the municipality does not have physical facilities to shelter them.

4. Is the provision in Article 16, Paragraph 4 of Municipal Law 13.908/2011, in verbis, fulfilled with regard to domestic animals such as dogs and cats? In what ways?

Article 16, Paragraph 4 of Municipal Law 13.908/2011 applies to those wild or feral animal species, native to the fauna of our city (“...released into their habitat...”) or exotic (“...delivered to zoos, foundations, sanctuaries and similar entities...”) under suitable conditions (“under the responsibility of a qualified technician and adapted to the receiving ecosystem”).

History of the Hoarder

Transparency and Sharing

In the early morning of Sunday, March 17, 2013, a serious fire occurred at the home of Mrs. Patient name, located at Patient address. Several complaints of animal abuse against Patient name had been filed, and several inspections were carried out at the location, as described below: As requested in Official Letter No. 2445/2010 - PP MPPR-0046.10.000299-0, Public Prosecutor's Office of the State of Paraná, an inspection was carried out on December 13, 2010. The person responsible for the property was not at home, and from the gate it was possible to verify the presence of approximately ten mixed-breed dogs, adults, males and females, in precarious hygienic conditions, with mud, a large amount of debris, food scraps, and animal feces. The dogs were found loose, chained, or sheltered in makeshift kennels made of wooden planks and recycled plastic bags. Some dogs lacked shelter to protect themselves from the cold, rain, or sun. The barking of other dogs could be heard, likely from inside the house. In response to the aforementioned official letter, new inspections were carried out on June 13, 2011, and July 20, 2011. In addition to the previously verified inadequate environmental conditions, approximately 40 (forty) mixed-breed dogs, adults and puppies, males and females, were found. Some dogs were thin, and others presented with generalized skin infections. According to the dogs' owner, there was no water supply from Sanepar (the water company) or electricity at the location. She received donations of water jugs from neighbors. The situation of the animals was discussed and analyzed in conjunction with the Municipal Health Department, through the Center for Zoonosis and Vector Control, and although it was found that the animals were being mistreated, the municipality did not have physical facilities to house them.

On January 9, 2012, a complaint of animal abuse was filed (protocol 004.092.697) regarding animals with mange and foul odor, with 80 dogs presenting mange and other diseases. An inspection was carried out, where the owner only mentioned 48 dogs and did not allow entry into the house. Animals with mange were observed, and treatment was scheduled for them.

In the complaint of July 25, 2012 (protocol 004.388.356), an inspection of animal abuse was requested, with injured dogs, with skin wounds and mange, and the resident abusing the dogs. The animals were medicated against mange and worms. The situation was monitored by the health surveillance. The person responsible was summoned by the Public Prosecutor's Office to arrange for the adoption of the animals.

On August 20, 2012, a complaint was filed regarding the case of Mrs. Patient name (protocol 004.425.992) alleging at least 80 animals with mange and other problems, causing discomfort in the neighborhood. The animals were treated for mange, worms, fleas, and ticks on August 22, 2012, and adoption was recommended.

In the animal abuse complaint dated November 8, 2012 (protocol 004.541.045), an inspection was carried out and the premises were found to lack hygiene and sanitation. Some of the animals had already been assisted by the Animal Defense and Protection Network, vaccinated, treated, and returned to Patient name, who refused to put the animals up for adoption. Given the situation, the protocol was forwarded to the Social Assistance Foundation (FAS) for assistance in approaching the resident.

In the animal abuse complaints dated January 9, 2013 (protocols 004.631.943 and 004.632.981) and January 21, 2013 (protocol 004652098), reporting the same inadequate conditions in which the animals lived, an inspection was carried out and a new notification was issued to the owner to improve the premises, promote the adoption of the animals, and not collect any new animals.

In parallel with these complaints, in May 2012 an injunction was issued ordering Ms. Patient name not to collect any more animals. Since August 2012, a Public Civil Action has been pending in the 23rd Civil Court, which is currently in the sentencing phase (case number 22917-72.2012.8.160001). Furthermore, on February 19, 2013, a request for an injunction was made for the removal of the animals against the owner's will. This injunction is awaiting a judicial decision. As per the aforementioned account, it can be inferred that patient actions are characteristic signs of animal hoarding syndrome, as described in the book *Clinical Forensics: Animal Cruelty Investigations*.

According to witnesses, in the early morning hours of the fire, the owner of the animals was using candles for lighting, as her residence was without electricity, which was the likely cause of the accident. Ms. Patient name did not provide assistance to the animals, which died of asphyxiation and were burned inside the residence, totaling 43 deaths. Twenty-nine dogs were rescued alive, including one female with eight puppies and 20 adult animals that were trapped outside the residence. These animals are under the care of the Amigo Animal Association, the Curitiba Animal Protection Society, and the Amigo de Raça animal protection group, and will be available for adoption at an opportune time.

REPORT OF VETERIAN OF CITY SECRETARY OF ENVIROMENT

I made the first visit in early 2018 (I don't have the exact date) at the address above. On this first visit, she herself told me about the previous episode, in which her house had been set on fire and about 50 dogs had died. She claimed that a candle started the fire. She stated that at the time she had 19 dogs, 9 females and 10 males, and 5 of them were 1 month old, still nursing. At that time, she received me, was polite, and despite the amount of swearing she used, she was not offensive. However, she never allowed me to enter the residence. The electricity had been cut off for non-payment, again posing a fire risk. She worked nights as a bartender, starting her shift around 6:00 PM and returning home around 3:00 AM.

She only allowed the spaying once, on which occasion 9 female dogs were scheduled, however, when we went to pick up the animals she only handed over 4 males that were neutered and microchipped at the Curitiba city hall's mass neutering event on 07/05/2018. The neutered dogs returned to her house on the same day.

She tried to divert our attention, always asking for our help from the neighbor who also had some dogs.

In early 2019 (I don't have the exact date), she left Curitiba, probably due to non-payment of rent, and went to live in São José dos Pinhais. At that time, the DPMA (Environmental Protection Police) was called and, during an inspection of the property in patient neighborhood, found 6 dead dogs in different stages of decomposition. I remember a news report at the time, but I couldn't find it.

In 2020, she was back in Curitiba and again with a large number of dogs, but her house was set on fire again on September 7, 2020, and 10 dogs died on that occasion, and the remaining dogs (I don't know the exact number) were rescued by animal rescuers. I found a link reporting this fact:

https://tnonline.uol.com.br/noticias/parana/morador-de-curitiba-e-preso-por-incendiar-casa-da-inquilina-10-caes-morreram-480485

Having assisted this lady, I suspect that this fire was not caused by the property owner, although I have no evidence of this fact.

After this second fire, she moved into her sister’s house. I contacted her on 09/05/2023 via WhatsApp and asked if Mrs. Patient name had acquired more dogs, and I offered free spaying and neutering and clinical action with vaccination. Her sister replied that she currently only has one female dog that was recently spayed by the city. She stated that her sister lives alone, but did not give me her current address.
